# Identification of Genes Involved in Bacteriostatic Antibiotic-Induced Persister Formation

**DOI:** 10.3389/fmicb.2018.00413

**Published:** 2018-03-06

**Authors:** Peng Cui, Hongxia Niu, Wanliang Shi, Shuo Zhang, Wenhong Zhang, Ying Zhang

**Affiliations:** ^1^Key Lab of Molecular Virology, Institute of Medical Microbiology, Department of Infectious Diseases, Huashan Hospital, Fudan University, Shanghai, China; ^2^Department of Molecular Microbiology and Immunology, Bloomberg School of Public Health, Johns Hopkins University, Baltimore, MD, United States; ^3^Lanzhou Center for Tuberculosis Research – Institute of Pathogenic Biology – Gansu Provincial Key Laboratory of Evidence Based Medicine and Clinical Translation, School of Basic Medical Sciences, Lanzhou University, Lanzhou, China

**Keywords:** persister, bacteriostatic antibiotics, antagonism, bactericidal antibiotics, persister mechanism

## Abstract

Persister cells are metabolically quiescent multi-drug tolerant fraction of a genetically sensitive bacterial population and are thought to be responsible for relapse of many persistent infections. Persisters can be formed naturally in the stationary phase culture, and also can be induced by bacteriostatic antibiotics. However, the molecular basis of bacteriostatic antibiotic induced persister formation is unknown. Here, we established a bacteriostatic antibiotic induced persister model and screened the *Escherichia coli* single gene deletion mutant library for mutants with defect in rifampin or tetracycline induced persistence to ofloxacin. Thirsty-seven and nine genes were found with defects in rifampin- and tetracycline-induced persister formation, respectively. Six mutants were found to overlap in both rifampin and tetracycline induced persister screens: *recA*, *recC*, *ruvA*, *uvrD*, *fis*, and *acrB*. Interestingly, four of these mutants (*recA*, *recC*, *ruvA,* and *uvrD*) mapped to DNA repair pathway, one mutant mapped to global transcriptional regulator (*fis*) and one to efflux (*acrB*). The stationary phase culture of the identified mutants and parent strain BW25113 were subjected to different antibiotics including ofloxacin, ampicillin, gentamicin, and stress conditions including starvation and acid pH 4.0. All the six mutants showed less tolerance to ofloxacin, but only some of them were more sensitive to other specific stress conditions. Complementation of five of the six common mutants restored the persister level to that of the parent strain in both stationary phase and static antibiotic-induced conditions. In addition to the DNA repair pathways shared by both rifampin and tetracycline induced persisters, genes involved in rifampin-induced persisters map also to transporters, LPS biosynthesis, flagella biosynthesis, metabolism (folate and energy), and translation, etc. These findings suggest that persisters generated by different ways may share common mechanisms of survival, and also shed new insight into the molecular basis of static antibiotic induced antagonism of cidal antibiotics.

## Introduction

Persister cells are a small population of metabolically quiescent bacteria that can tolerate lethal antibiotic treatment (Hobby et al., 1942; Bigger, 1944). Unlike resistant mutants, which have some modification(s) in the genetic material and still can grow in the presence of antibiotics, persistence is a general phenomenon for all bacteria due to epigenetic changes that do not involve any mutations, though some mutations could change the frequency of persisters (Lewis, 2007). Although persisters do not grow in the presence of antibiotics, they remain alive and can still be killed by the antibiotic when re-inoculated to fresh medium (Lewis, 2007). Persisters were found more than 70 years ago (Hobby et al., 1942), but they were overshadowed by resistance for a long time, and got more attention recently. Persisters are considered responsible for relapses in many persistent infections such as tuberculosis (Zhang, 2004; Zhang et al., 2012), urinary tract infection (Blango and Mulvey, 2010), cystic fibrosis (Lyczak et al., 2002), Lyme disease (Stricker et al., 2011), and biofilm infection of indwelling devices (Stewart, 2002).

Persister cells in a log phase culture are rare, ranging from 0.001 to 0.01% of all bacteria, but in stationary phase cultures, the persister frequency can increase up to 1% (Lewis, 2007). How the persisters increase in numbers and what makes them survive the antibiotic treatment are still unclear. One theory is that persisters are derived from stochastic changes in gene expression that cause dormancy and low metabolic activity (Lewis, 2000), but increasing evidence demonstrates that persisters are under delicate control by environment (Vega et al., 2012; Conlon et al., 2016; Harms et al., 2016; Kim et al., 2016). TA (toxin-antitoxin) systems are the most intensively researched mechanism of persister formation in *E. coli*. *hipA* is the toxin of the *hipAB* TA system. *hipA7*, a gain of function mutation of *hipA* but is not toxic, is the first gene found involved in persister formation (Moyed and Bertrand, 1983). HipA overexpression enhanced phosphorylation of GltX, leading to accumulation of uncharged tRNA-Glu, which in turn activates the stringent response causing a high level of persisters (Kaspy et al., 2013). Other TA systems like MqsR/MqsA (Kim and Wood, 2010), RelB/RelE (Tashiro et al., 2012), and TisB/IstR-1 (Dorr et al., 2010) are also involved in persister formation. Besides the TA system, many other persister mechanisms are found in *E. coli*, including metabolic genes such as *glpD* (Spoering et al., 2006), *sucB* and *ubiF* (Ma et al., 2010), SOS response gene *recA* (Dorr et al., 2009), stringent response gene *relA* (Korch et al., 2003), phosphate metabolism gene *phoU* (Li and Zhang, 2007), and *trans*-translation (Li et al., 2013). This indicates that persisters are formed through highly redundant mechanisms (Zhang, 2014; Harms et al., 2016).

It is well known that bacteriostatic antibiotics can antagonize the killing activity of the cidal antibiotics (Jawetz et al., 1950; Brown and Alford, 1984; Ocampo et al., 2014). For example, bacteriostatic agent chloramphenicol could antagonize the activity of bactericidal agent penicillin (Jawetz et al., 1950; Brown and Alford, 1984). In addition, quinolones have activity against both growing and non-growing bacteria, but when ofloxacin or ciprofloxacin (two bactericidal quinolone antibiotics) was combined with static antibiotic chloramphenicol, the percentage of surviving *Staphylococcus aureus* cells was at least two orders of magnitude higher than the quinolone treatment alone (Lewin and Smith, 1988). Recently, it has been shown that static antibiotics inhibiting transcription and translation increased the persister frequency significantly through inducing a quiescent state (Kwan et al., 2013). These data imply that in the presence of bacteriostatic antibiotics, the bacteria turn into persisters, but the mechanisms by which static antibiotics induce persister formation are unknown. To provide insight into the molecular basis of bacteriostatic antibiotic induced persisters, we screened the *E. coli* gene knockout mutant library (Keio collection) (Baba et al., 2006), to identify mutants that can still be killed by ofloxacin in the presence of bacteriostatic antibiotics rifampin or tetracycline. We found that in addition to genes encoding DNA repair pathway (*recA*, *recC*, *ruvA*, *uvrD*) other genes coding for transcription factor (*fis*), transporters, LPS biosynthesis, flagella biosynthesis, metabolism (folate and energy), translation, and some genes of unknown function are necessary for bacteriostatic agent-induced persister formation.

## Materials and Methods

### Antibiotics, Strains, and Culture Conditions

Ofloxacin, kanamycin, rifampin, tetracycline, and gentamicin were obtained from Sigma-Aldrich Chemical Co., and were used at 5, 30, 100, 50, 20 μg/ml, respectively. The *Escherichia coli* single gene deletion mutant library Keio collection and the parent strain BW25113 were used for the screen (see below). All experiments were conducted at 37°C in Luria-Bertani (LB) medium.

### Screening of the Keio Mutant Library

The *E. coli* Keio mutant library which contains 3985 individual deletion mutants arrayed in forty-six 96-well plates was grown in 200 μl fresh LB medium containing 30 μg/ml kanamycin overnight at 37°C without shaking. The mutant library was then transferred to fresh LB medium again and grown for 4 h before bacteriostatic agent rifampin (RIF) (100 μg/ml) or tetracycline (50 μg/ml) was added to each well to induce the formation of persister cells (Kwan et al., 2013).

In pilot experiments, we found that 30 min induction with RIF or tetracycline was sufficient to observe the static agent induced increase in persisters to cidal drug ofloxacin, and thus ofloxacin was added to the library 30 min after the static antibiotic treatment. The mutant library was incubated for 3 days further, and then replicated to LB agar plates using metal 96-well pin replicator (Sigma R2508). After incubation overnight, the wells with no or poor growth were chosen for further examination. The plate treated with ofloxacin alone was set as a control.

### DNA Manipulation and Plasmid Construction

To complement the gene knockout mutants picked from the Keio mutant library, the entire open reading frame (ORF) and its native promoter (about 200 bp upstream of ORF) of the candidate gene was amplified by polymerase chain reaction (PCR) and then ligated to the pCR2.1 TA-cloning vector per the manufacturer’s protocol (Invitrogen, Carlsbad, CA, United States). The plasmids were extracted using the Pure-Link^TM^ Quick Plasmid Miniprep Kit (Invitrogen, Carlsbad, CA, United States) and sequenced to confirm the right inserts. Online tool https://ecocyc.org/ was used for promoter prediction and the information was used to construct complementation plasmids. The complementation plasmids and empty vector were then transformed into the corresponding mutants.

Primers used to amplify the candidate genes were as follows: *fis*: 5′-TGGACA CTGGGGAGTTGCTG-3′ and 5′-GTCGGTTCACATCCTGTTCTCAT-3′, *recA*: 5′-GTGCTGATTATGCCGTGTCTATTA-3′ and 5′-CCGCAGATGCGACCCTTG-3′, *recC*: 5′-TGCGTTATCGGGTTTCCAG-3′ and 5′-TGCACGAGTCAGCC TATGTTTAT-3′, *ruvA*: 5′-CGTTGTCATTCCATTGAAATAGATAC-3′ and 5′-TGCGGCTGACCAACATACTC-3′, *uvrD*: 5′-AATTCGCAGCGGAATGC-A-3′ and 5′-CGA GCGGAAAGGTTAAAACG-3′.

### Susceptibility to Antibiotics and Various Stresses

Stationary phase overnight cultures of the mutants, complemented strains and *E. coli* parent strain BW25113 were exposed to different antibiotics such as ofloxacin (5 μg/ml), ampicillin (100 μg/ml), and gentamicin (20 μg/ml). At different time points, 100 μl of the culture was removed and washed with phosphate buffered saline (PBS) and then serially 10-fold diluted and plated for colony formation unit (CFU) counts. For starvation and acid stress assay, overnight cultures were washed and diluted 100-fold in saline or pH 4.0 LB, respectively, and incubated at 37°C without shaking for different time points followed by CFU determination.

### Statistical Analysis

The significance of experimental differences between the mutants and parent strain BW25113 in persister assays was evaluated by unpaired Student’s *t*-test.

## Results

### Pre-treatment With Rifampin Significantly Increased Persister Numbers

**FIGURE 1:**
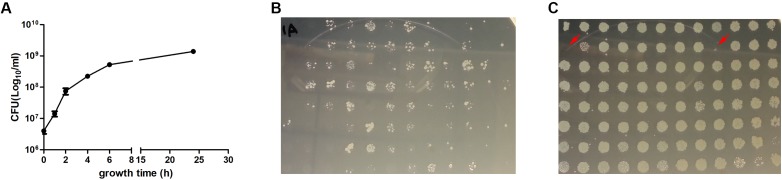
Screens for mutants with defect in bacteriostatic antibiotic induced persister formation. Growth curve of six randomly chosen mutants in 96-well plates, mean and standard error of the mean was shown **(A)**. For the pilot screen, one plate of the Keio library was inoculated to fresh LB broth duplicate, grown for 4 h to log phase and treated for 3 days with 5 μg/ml ofloxacin **(B)**, or pretreated with 100 μg/ml rifampin for 30 min and then added 5 μg/ml ofloxacin for 3 days **(C)**. The plates were then replicated to LB agar and grown overnight at 37°C. The clones with red arrow were considered as positive and selected for further analysis.

In the model of bacteriostatic antibiotic induced persisters established by Kwan et al. (2013), the static antibiotics were added to the log phase culture for 30 min and washed away before adding the cidal antibiotics. In another similar work, the static antibiotic was added with the cidal antibiotic simultaneously and similar results were obtained (Lewin and Smith, 1988). As we intended to screen the Keio mutant library which contains forty-six 96-well plates, it was more realistic to leave the static antibiotics in the well when adding the cidal antibiotic, especially when prior studies have shown that removing or keeping the static antibiotic did not make any difference (Lewin and Smith, 1988; Kwan et al., 2013). We first did a growth curve in 96-well plates by randomly choosing six clones from the plate and determined viable CFU over time. The results showed that the cells entered late log phase when cultured for 6 h (**Figure 1A**). We then tested different growth times and drug exposure times to optimize the screen method. The 96 pin replicator can transfer about 0.5–1 μl out of 100 μl culture in the 96-well plates onto LB agar, and the culture transferred contained small amount of antibiotics, in the case of 5 μg/ml ofloxacin, the antibiotic would decrease the viable cells by another order of magnitude (data not shown), so the detection limit for this method is about 2 × 10^4^ CFU/ml. When the library was grown for less than 2 h, then exposed to ofloxacin for 1 day, and finally replicated onto LB agar, no colonies were recovered on the plates, whether the bacteria were pretreated with RIF or not. When the culture was older than 8 h, ofloxacin alone and ofloxacin plus rifampin could not kill the bacteria in 3 days (data not shown). We optimized the screening procedure by growing the Keio mutant library for 4 h, added the static antibiotics for 30 min to induce persisters and then added ofloxacin, and 3 days later replicated the 96-well plates onto LB agar plates. In this condition, the ofloxacin alone could kill nearly all the bacteria (**Figure 1B**) while the ofloxacin combined with rifampin was much less effective, indicating an antagonism by the bacteriostatic agent rifampin against the cidal agent ofloxacin (**Figure 1C**). This demonstrates that rifampin pretreatment indeed increased the persister frequency. Although most of the mutants still had robust growth after treatment with rifampin plus ofloxacin, some mutants showed no or little growth in the plate. These mutants were picked for further rescreens and analyses (see below).

### Pathways Involved in Rifampin Induced Persisters

**FIGURE 2:**
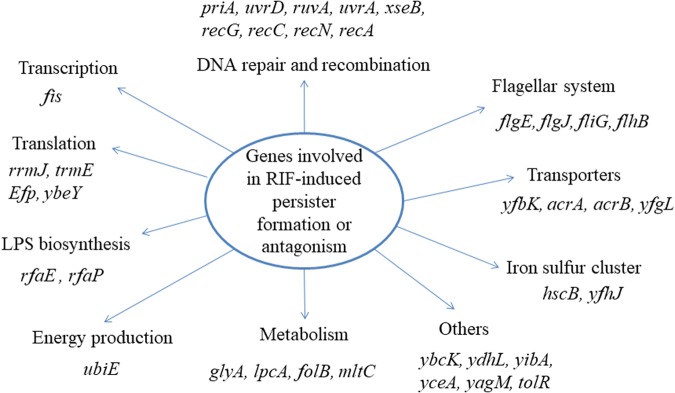
Pathway analyses of the 37 gene deletion mutants whose genes participate in the rifampin induced antagonism or persister formation to ofloxacin.

Using the method established above, we screened the Keio mutant library with bacteriostatic agent rifampin plus ofloxacin in comparison with a control screen with ofloxacin alone without rifampin. The target strains were repeated at least twice to confirm a stable phenotype. Thirty seven mutants (*priA, uvrD, ruvA, uvrA, xseB, recG, recC, recN, recA, flgE, flgJ, fliG, flhB, yfbK, acrA, acrB, tolR, yfgL, fis, glyA, ubiE, rfaP, lpcA, folB, rfaE, mltC, rrmJ, trmE, efp, ybeY, hscB, ybcK, ydhL, yibA, yfhJ, yceA, yagM*) were found to lose the phenotype of rifampin induced persister formation or antagonism (**Figure 2**). Interestingly, many of them are involved in DNA repair and recombination pathway, such as *recA*, *recC*, *recN*, *recG*, and *ruvA*. This pathway is closely related to the killing mechanism of quinolones, which can cause double strand DNA breaks. In addition to the DNA repair pathway, genes involved in metabolism (*glyA, lpcA, mltC*), translation and transport (*rrmJ, trmE, efp, ybeY, acrA, acrB*), LPS biosynthesis (*rfaP, rfaE*), flagella biosynthesis (*flgE*, *flgJ*, *fliG* and *flhB*), and genes of unknown function (*ybcK, ydhL, yibA, yfhJ, yceA, yagM*), were also found to be involved in rifampin-induced persister formation.

### Screening the Keio Mutant Library Pretreated With Tetracycline

To determine if there is a common mechanism of bacteriostatic agent induced persister formation, we screened the Keio mutant library with another static antibiotic tetracycline in treatment with ofloxacin. Unlike rifampin, which binds to RNA polymerase and inhibits mRNA synthesis (Campbell et al., 2001), tetracycline binds to the 30S subunit of bacterial ribosomes and inhibits protein synthesis (Chopra and Roberts, 2001), and was used as a pretreatment drug to screen the Keio mutant library treated with ofloxacin. After 3 days of drug exposure, nine mutants showed defect in conferring tetracycline induced antagonism or persister formation against ofloxacin. These nine mutants mapped to *acrB*, *fis*, *recA*, *recC*, *ruvA,* *uvrD*, *rpoN*, *yfaD* and *dksA*. The first six of the nine genes were also found in the above screen with rifampin, which demonstrated a high degree of conservation of the mechanisms involved, whereas *rpoN*, *yfaD*, and *dksA* are unique genes identified to be involved in tetracycline-induced persister formation.

### Time Dependent Killing Curve of the Mutants

**FIGURE 3:**
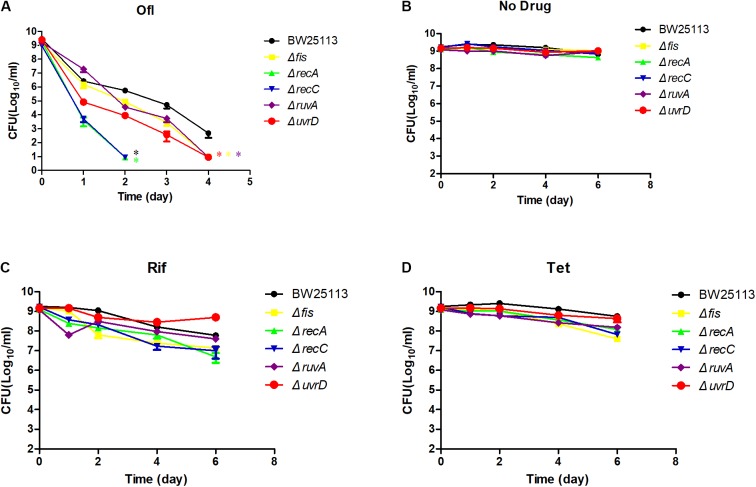
Time-dependent killing of selected mutants by ofloxacin and static antibiotics in stationary phase culture. Five of the common mutants in the rifampin and tetracycline induced antagonism screens and the parent strain BW25113 were grown overnight and then treated with 5 μg/ml ofloxacin **(A)**, no drug control **(B)**, rifampin **(C)**, and tetracycline **(D)**. At different time points of incubation, bacterial survival was determined by CFU count after removal of the antibiotic by washing. Data represents three independent biological replicates. Error bars represent standard error of the mean (SEM). ^∗^Indicates a statistical difference between mutants and parent strain (*P* < 0.05, *t*-test).

To more precisely evaluate the effect of gene deletions on persister formation, the common mutants except *acrB* along with parent strain *E. coli* BW25113 were subjected to persister assays. All the mutants were confirmed by PCR using primers adjacent to their ORFs (Supplementary Table S1). We first conducted an MIC experiment for ofloxacin, rifampin, and tetracycline with these mutants (**Table 1**). All the mutants had the same MIC for tetracycline, and *ruvA* and *recC* mutants showed a twofold decrease of MIC for rifampin. But for MIC to ofloxacin, the *fis* and *ruvA* mutants showed a 2- and 4-fold decrease, respectively, while *recA* and *recC* mutants had nearly 10-fold decrease. To assess persister formation, stationary phase cultures were used because the persister frequency is much higher than that of log phase cultures. All of the included mutants were more susceptible to ofloxacin than the parent strain BW25113 (**Figure 3A**). *recA* and *recC* mutations showed significant changes in survival even after only 2 days of antibiotic exposure (*P* < 0.05), and the surviving cells in the two mutants were below the detection limit (10^1^ CFU/ml), whereas more than 10^5^ CFU/ml bacteria still remained in the parent strain BW25113. Depending on various time points, the *fis*, *ruvA* and *uvrD* mutants exhibited 10- to 1000-fold decrease in persistence levels compared with the parent strain. All of these mutants showed significant difference with the parent strain at day 4 time point (*P* < 0.05). The no drug control indicated that the bacterial number was not decreased during the time span of the experiment (**Figure 3B**), and all the strains had no significant differences in susceptibility to rifampin and tetracycline (*P* > 0.05) (**Figures 3C,D**).

**Table 1:** The MICs of the mutants and parent strain *E. coli* BW25113 to different antibiotics.

	MIC (mg/L)		
Strains	Ofloxacin	Rifampin	Tetracycline
BW25113	0.05	6.25	1.2
Δ*fis*	0.025	6.25	1.2
Δ*ruvA*	0.012	3.12	1.2
Δ*recA*	0.006	6.25	1.2
Δ*recC*	0.006	3.12	1.2
Δ*uvrD*	0.05	6.25	1.2

### Susceptibility to Different Classes of Antibiotics and Stresses

**FIGURE 4:**
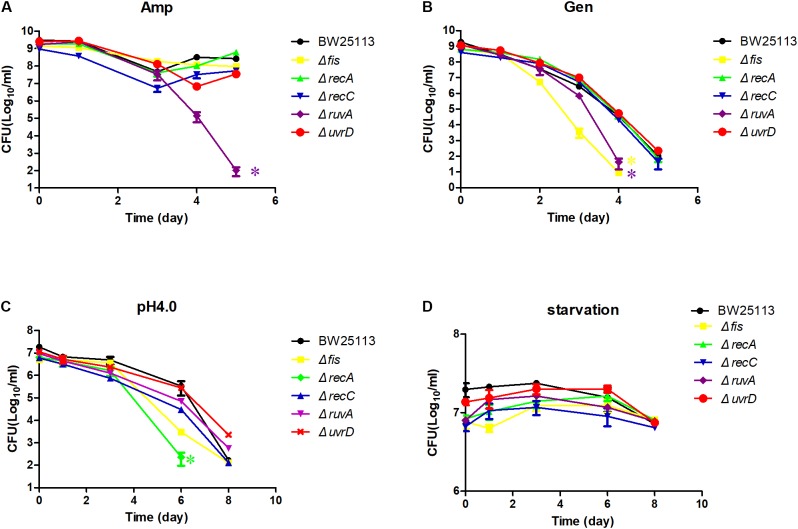
Susceptibility of the mutants to various antibiotics and stress conditions. Stationary phase culture of the mutants and the parent strain BW25113 were treated with ampicillin **(A)**, gentamicin **(B)**, washed and diluted 100-fold to acid pH 4.0 LB broth **(C)**, washed and diluted 100-fold in 0.9% saline for starvation **(D)**, followed by CFU determination on LB agar plates at different time points. Data represents three independent biological replicates. Error bars represent standard error of the mean (SEM). ^∗^Indicates a statistical difference between mutants and parent strain (*P* < 0.05, *t*-test).

We next exposed the identified mutants to other antibiotics in addition to quinolone drug, such as ampicillin, gentamicin, and also more stress conditions such as starvation and acid pH (**Figure 4**). For the ampicillin exposure, there was only a slight difference between the mutants and the parent strain BW25113, except *ruvA* mutant (*P* < 0.05) (**Figure 4A**). All the mutants except *uvrD* were more susceptible to gentamicin, but only *fis* and *ruvA* mutants had the strongest persister defect phenotype, dropping about 1000-fold in CFU count compared with the parent strain at day 4 (*P* < 0.05) (**Figure 4B**). When the mutants were diluted 100-fold to acid pH 4.0 LB, the persister levels of the *recA* was greatly reduced more than 100-fold compared with the parent strain BW25113 at day 6 (*P* < 0.05) (**Figure 4C**), while the *uvrD* and *ruvA* mutants showed slightly more tolerance to the acid stress than the parent strain. The difference in persister levels between the mutants and the parent strain was less obvious when they were exposed to the starvation condition in saline (*P* > 0.05) (**Figure 4D**).

### Complementation of the Mutants Restored the Static Antibiotic Induced Persisters

The screen and persister assay revealed that the mutants were less tolerant to different antibiotics and stress conditions, especially ofloxacin. Five of the mutants *recA*, *recC*, *uvrD, ruvA* and *fis* were complemented to further characterize their ability to confer persistence. The genes’ own native promoters were used instead of an inducible promoter to avoid overexpression, which may cause non-specific toxicity (Vázquez-Laslop et al., 2006).

**FIGURE 5:**
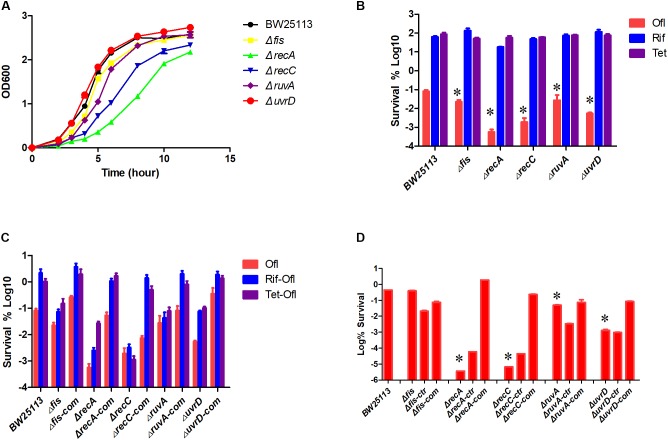
Complementation of the mutants restored the persistence phenotype. Growth curve of the parent strain BW25113 and five mutants **(A)**. Sensitivity of the log-phase culture to ofloxacin, rifampin, and tetracycline **(B)**. Log phase culture of selected mutants, complement strain and parent strain treated with ofloxacin or pretreated with rifampin or tetracycline for 30 min and then treated with ofloxacin for 3 h. Surviving cells were counted by serial dilution and inoculation on LB plates **(C)**. All the mutants and complement strains were cultured to stationary phase and then treated with ofloxacin for 2 days **(D)**. -ctr: empty plasmid; -com: complemented with corresponding genes. Data represents three independent biological replicates. Error bars represent SEM. Data represents three independent biological replicates. Error bars represent standard error of the mean (SEM). ^∗^Indicates a statistical difference between mutants and parent strain (*P* < 0.05, *t*-test).

To avoid the impact of different growth rate on persister levels, especially the growth defect of the *recA* mutant (**Figure 5A**), we used the bacteria at a concentration of about 2 × 10^8^ CFU/ml (OD_600_ around 0.3). We first tested the susceptibility of the log-phase culture of these strains to the antibiotics used during the screen, and the results showed that the mutants were killed more rapidly by ofloxacin but not by rifampin and tetracycline (**Figure 5B**). The persister levels in the log phase cultures induced by bacteriostatic antibiotics were also measured for the complemented strains (**Figure 5C**). Pretreatment with either rifampin or tetracycline increased the persister levels at least 10-fold in BW25113, demonstrating their persister inducing capability. In the case of *recC* gene, the gene deletion mutant exhibited the same persister level whether there was static antibiotic or not (*P* > 0.05), but the complemented strain restored two important features: a higher persister level to ofloxacin compared with the gene deletion mutant, which indicates restoration of persistence ability, and a higher persister level when pretreated by static antibiotics than without pretreatment. Log phase *ruvA* mutant showed no difference in persister level when pretreated by static antibiotics (*P* > 0.05), while the complementation restored it to the level of the parent strain.

*fis, uvrD,* and *recA* deletion mutant also had the static antibiotic inducible persister phenomenon as BW25113 but at a much lower persister level, and the complemented strain reached the persister levels of the parent strain at all conditions tested.

Overnight stationary phase cultures of these mutants and the mutants transformed with empty vector still showed a defect in persistence to ofloxacin, while the persister levels of their respective complemented strains were restored to that of the parent strain BW25113 (**Figure 5D**).

## Discussion

It is well known that bacteriostatic agents can antagonize the activity of bactericidal antibiotics when used together. The mechanism behind this observation is not well understood. In this study, we first established the bacteriostatic agent induced antagonism model using rifampin and tetracycline and then screened the entire single gene deletion mutant library of *E. coli* to identify the molecular basis of this phenomenon. We found deletion mutants with defect in genes that belong to DNA repair and recombination pathway, such as *recA*, *recC*, *ruvA*, and *uvrD* to be most prominent among the genes identified. Although the Keio library was used previously to find persister related genes, this is the first time it is used for identifying bacteriostatic antibiotic induced antagonism of bactericidal antibiotics or persister formation. The high consistency of our screen results with two different classes of static antibiotics demonstrates the reliability of our results and also suggests a common mechanism underlying the static antibiotic induced antagonism and persister formation. Besides the DNA repair pathways shared by both rifampin and tetracycline induced persisters, we identified new genes involved in transporters, LPS biosynthesis, flagella biosynthesis, metabolism (folate and energy), translation, and genes of unknown function, that are specific to rifampin-induced persisters.

Among the six common genes identified by both screens, most have been found to participate in stationary phase induced persister formation. AcrB is a component of AcrAB-TolC efflux pump that extrudes a wide variety of drugs and toxic compounds, and its mutation may affect the transport of ofloxacin, leading to a higher drug concentration in the cell and a lower persister level (Wu et al., 2012). Both the *recA* and *recC* belong to SOS-regulon that are essential for the repair of double DNA breaks (Dillingham and Kowalczykowski, 2008), which is the major lethal effect of quinolones (Drlica et al., 2008). The *recA* and *recB* mutations have been found to cause decrease in persister numbers when challenged with ciprofloxacin in an exponential growth phase culture (Dorr et al., 2009), and were required for persister formation under ofloxacin treatment due to their involvement in the recovery phase (Völzing and Brynildsen, 2015). However, *recC* is not previously described as a persister gene, and we found that its mutation has the most profound effect in causing decrease in persister numbers in our study. RecC together with RecB and RecD constitutes exonuclease V, which initiates recombination repair of double strand breaks (DSBs) in DNA. RecC is critical for the recognition of Chi sites and the regulation of exonuclease activity of RecB. It is possible that RecBCD enzyme involved in DSB may play a more important role than RecA involved in single strand break and recombination during the ofloxacin treatment. Mutations in the other two DNA repair genes *ruvA* and *uvrD* were previously shown to have less tolerance to ciprofloxacin in the exponential phase culture (Theodore et al., 2013). RuvA binds Holliday junctions with high affinity, and stimulates the ATPase activity of RuvB. RuvABC complex mediates branch migration and resolves the Holliday junction during homologous recombination (West, 2003). UvrD is a DNA helicase with DNA-dependent ATPase activity. It has an important role in DNA mismatch repair, nucleotide excision repair, and recombinational repair (Lee and Yang, 2006). Fis is a global transcription regulator that controls more than 200 genes, including genes involved in energy metabolism, flagellar synthesis, protein synthesis and carbon metabolism (Bradley et al., 2007). The *fis* deletion mutant showed a strong persister deficiency in the screen, but when in a larger volume of culture, the persister level of the *fis* mutant was not different compared to the parent strain. This phenomenon was reported before (Hansen et al., 2008), but the reason is not clear. Although some of these mutants showed a decreased MIC, we think that they should not be excluded from the persister genes, because MIC reflects the ability of the antibiotic to inhibit bacterial growth while the persister assays reflect the ability of the antibiotic to kill the bacteria. While the *recA*, *recC,* and *ruvA* mutants had a decreased MIC to ofloxacin, the more susceptibility of these mutants to the cidal antibiotic could also contribute to their faster killing by the antibiotic treatment.

Besides the common genes of two different screen conditions, more genes were found when pretreated with rifampin than with tetracycline. The reason for this phenomenon is not known, but one possibility is the growth variations of different batch of culture. To avoid the tedious washing and dilution of the library after drug exposure, and to observe the deep persisters, we chose a 3-day exposure to ofloxacin, a time that the most sensitive mutants cannot form colony on the plate after the drug exposure. Another study was done using the same mutant library at stationary phase culture exposed to ofloxacin for just 6 h (Hansen et al., 2008), in contrast to 3 days as in this study. Comparison with that work found many overlapping genes. Apart from many DNA repair and recombination pathway genes which were excluded from the previous study due to a changed MIC, the transcription regulators *fis* and *dksA* (rRNA transcription regulator) were identified in both screens.

The flagella synthesis genes (*flgE, flgJ, fliG, flhB*) unexpectedly appeared in rifampin induced antagonism screen, and to our knowledge, these genes were not known to be related to persister formation. Other genes identified in the rifampin induced antagonism or persister formation experiment are those involved in transporters (*yfbK, acrA, acrB, yfgL*), LPS biosynthesis (*rfaEP*), metabolism (*glyA, lpcA, mltC*), translation (*rrmJ, trmE, efp, ybeY*), etc. Mutations in LPS biosynthesis genes *rfaP* and *rfaE* would cause changes in permeability of the cell envelope and could allow antibiotic ofloxacin to enter more readily (Liu et al., 2010). GlyA catalyzes the interconversion of serine and glycine with tetrahydrofolate (THF) as the one-carbon carrier, which is required for the biosynthesis of thymidylate, methionine, purines, and other important molecules necessary for metabolism. In addition, *folB*, which encodes dihydroneopterin aldolase that converts 7,8-dihydroneopterin to 6-hydroxymethyl-7,8-dihydropterin, is necessary for biosynthesis of folate cofactors. Folate in turn is needed for synthesis of thymidine triphosphate (dTTP), and defects in dTTP synthesis could cause thymine-less death in bacteria. Consistent with this finding is our previous observation that *uvrA, relA,* and *sucB* mutants are more susceptible to static agent trimethoprim, an inhibitor of folic acid synthesis (Wu et al., 2015).

We also tested the susceptibility of the identified mutants to other antibiotics and stress conditions. Only some of the mutants were more susceptible to a particular condition. For example, the *fis* and *ruvA* mutants were less tolerant to gentamicin, while the *recA*, *uvrD,* and *recC* mutants nearly had the same killing curve as the parent strain BW25113 (**Figure 4B**). However, in pH4.0 acid stress, the *recA* mutant had the most significant decrease in persister numbers, but intriguingly, the *uvrD* mutant survived even better at acid pH than the parent strain BW25113 (**Figure 4D**). The only condition that all the mutants were more susceptible than the parent strain was exposure to ofloxacin, the screening condition. This result demonstrated that the mechanism leading to bacterial death in different antibiotic treatment or stress conditions may not be the same and that the bacteria may need different gene(s) to survive under each specific condition. Our results are in accordance with the previous data that the persisters are highly heterogeneous (Li and Zhang, 2007; Allison et al., 2011; Goneau et al., 2014). Complementation experiments confirmed that the persister defect was indeed caused by the genes identified.

In summary, we showed that persisters generated by static antibiotics share similar survival mechanisms as those formed naturally in stationary phase due to their shared pathways. In addition to the DNA repair pathways shared by both rifampin and tetracycline induced persisters, other genes mapped to transporters, membrane biogenesis, LPS biosynthesis, flagella biosynthesis, metabolism (folate and energy), and translation, are more specific to rifampin-induced persisters. Future studies are needed to determine if these identified genes also play a role in the more complex *in vivo* environment induced persister survival.

## Author Contributions

YZ, PC, and WZ designed the experiments. PC, HN, WS, and SZ performed the experiments. PC, WZ, and YZ analyzed the data. PC and YZ wrote the manuscript.

## Conflict of Interest Statement

The authors declare that the research was conducted in the absence of any commercial or financial relationships that could be construed as a potential conflict of interest.
