# Supplementary material for: Identification of Genes Involved in Bacteriostatic Antibiotic-Induced Persister Formation
Source: Front Microbiol. 2018 Mar 6;9:413. doi: 10.3389/fmicb.2018.00413 (PMC5845583; doi:10.3389/fmicb.2018.00413)
Supplement: Supplementary file 1 [file Table_1.DOC]

Table S1 Primers used to confirm the mutants from the Keio library.

| **Primer name** | **Sequence** |
| --- | --- |
| recA F | GCATCTGCGGCCCTTTT |
| recA R | CCAGCTCCAGCGTGTCTTAA |
| recC R | ATTCAACACGTTAACGCAAATCATCT |
| recC F | CGTCAGTAGTCAGGAGCCGC |
| fis F | TGCGTAAACAGAAATAAAGAGCTGACAGAA |
| fis R | CCTTTTTAATCAAGCATTTAGCTAACCTG |
| uvrD F | CCTATTTTTACGCGGCGGTG |
| uvrD R | CAGCACCGCATCCGGCAA |
| ruvA R | ACGGTCTGCTTCAATCATCCTTTA |
| ruvA F | CTTTGATTCATTACGCAGGAGCG |
| dksA R | TAAACGTGATGGAACGGCTG |
| dksA F | AGTGCGTGTTAAGGAGAAGCA |
| xseB R | CGAGTTGCTGCGGAAAGTC |
| xseB F | GGCGAAAAATATTGAGAGTCAGACATTC |
| acrB R | GCGGCCTTAGTGATTACACGT |
| acrA R | GCATGTCTTAACGGCTCCTGTT |
| acrB F | AGTCTTAACTTAAACAGGAGCCGTTAAG |
| acrA F | TTGAAATCGGACACTCGAGG |
| tolR F | TTAGCGAGAGCAACAAGGGGTA |
| tolR R | AGCAAGGGAAACGCAGA |
| flgE F | GCCTTCACATTTCAGGAGTCAG |
| flgE R | CATTGAGCTATCCCGTCAGC |
| flgJ F | TGCGGGCAAAACTGGAAATCAT |
| flgJ R | AGCGACCCGCCGGACTT |
| flhB R | GGCAGGCGCAGCATCGC |
| flhB F | CGCTTAATACTCTTTCCAGGATTGG |
| fliG F | GCTGGTCATTCGCCAGTGG |
| fliG R | GTCCAGGTTTTCCACGGCAG |
| yfaD F | GCGTCATCATAAATATCAGGTGACGGA |
| yfaD R | CCGTTCAGCAATAGTCATTAATCTCTCC |
| glyA R | ACATTGACAGCAAATCACCGTT |
| glyA F | TTGTTAGCTGAGTCAGGAGATGC |
| recN F | AGTAATGGTTTTTCATACAGGAAAACG |
| recN R | GCTGTTTACTCTGACCGTGAAGCA |
| folB R | ACCGTTTGGTTTAACAGCTGT |
| folB F | GCCAAAAAGCAGGCAGGAC |
| rpoN F | GTTTTAGCAGGAGAGTACGATTCTG |
| rpoN R | TGCATAGTGTCTTCCTTATCGGTTG |
| recG F | ATGCAGGCTGCAGGGTAAGT |
| recG R | GCGGTCTTCTCACTGCCG |
| ubiE F | GGAACAATTTTTTGATGAGCAGGCA |
| ubiE R | AAAAGGCATTTCCGGTCTCC |
| priA R | GATCCGCCTCGCATCGTGA |
| priA F | CCACCGAATTTCAAGTCAGGATGAT |
| lpcA F | CATTTTGTCTATTACATTTATGCTGAAGGA |
| lpcA R | ACGTCTTATCCGGCCTACGCC |
| yagM R | TCTCATAATTGTTTGTCCTTCTTTGT |
| yagM F | TTGAGATAACAAAGAGGTTTCC |
| ydhL R | ATGAGAGGAGTATACGCAAGATTA |
| ydhL F | ATAATGGTAATAATCTTATCTGAGGAGGG |
| hscB R | GGCCATGTTTAGCTTCCAGAA |
| hscB F | CGGATCGCAGCCCTGAGAA |
| mltC F | ACACAACACGCACCCGGA |
| mltC R | AGCATCGTCAGGGGCGGT |
| rfaE R | GAAGCGAGATCTGTGAACCGC |
| rfaE F | CGCGCAAATTTTGAATCTCTCAGGAG |
| rrmJ R | CTTTCAAACTTTCGTCTGAAATCTCC |
| rrmJ F | ACGATGAGTTATCCCCATGGGAAA |
| rfaP | AATAAAGTTAGTTCCAGTACATACTAATAAA |
| rfaP F | CCGCGGATATCATTACAGGT |
| trmE F | TCCGCCACACAAAGCAACAGG |
| trmE R | ATGCGGCTTCGTAAGCG |
| efp F | ACCAATTAACAAATTTCAGAGGGC |
| efp R | TGCAGGCCGCACCACAACC |
